# Prediction of precise subsoiling based on analytical method, discrete element simulation and experimental data from soil bin

**DOI:** 10.1038/s41598-021-90682-w

**Published:** 2021-05-26

**Authors:** Nelson Richard Makange, Changying Ji, Innocent Nyalala, Idris Idris Sunusi, Samwel Opiyo

**Affiliations:** 1 College of Engineering, Nanjing Agricultural University, Nanjing, 210031 China; 2 Department of Engineering Sciences and Technology, Sokoine University of Agriculture, P.O.BOX 3003, Morogoro, Tanzania

**Keywords:** Engineering, Software

## Abstract

Prediction of a precise subsoiling using an analytical model (AM) and Discrete Element Method (DEM) was conducted to explain cutting forces and the soil profile induced changes by a subsoiler. Although sensors, AMs and DEM exist, there are still cases of soil structure deformation during deep tillage. Therefore, this study aimed to provide a clear understanding of the deep tillage using prediction models. Experimental data obtained in the soil bin trolley with force sensors were used for verification of the models. Experiments were designed using Taguchi method. In the AM, the modified-McKyes and Willat and Willis equations were used to determine cutting forces and soil furrow profile respectively. Calculations were done using MATLAB software. The elastoplastic behavior of soil was incorporated into the DEM. The DEM predicted results with the best regression of 0.984 $R^{2}$ at a $NRMSE$ of 1.936 while the AM had the lowest $R^{2}$ of 0.957, at a $NRMSE$ of 6.008. All regression results were obtained at *p* < 0.05. The ANOVA test showed that the p-values for the horizontal and vertical forces were 0.9396 and 0.9696, respectively. The DEM predicted better than the AM. DEM is easy to use and is effective in developing models for precision subsoiling.

## Introduction

Precision agriculture is modern farming management which is a technology-enabled method that detects, measures and examines the needs of individual fields. It uses digital techniques to monitor and optimize agricultural production processes. This brought up the need for smart agricultural machinery which are manufactured with so many sensors to acquire the precise data measured. However, it leads to high operation costs and sometimes low efficiency in precision control. To make the electro-hydraulic control in the tillage operation more precise, engineers need to know the force distribution of the tillage tools reacting on the three-point hitch. Therefore, instead of applying an equal amount of cutting force during tillage operation in every field, precision tillage involves measuring the within-field soil strength variations and apply to the field accordingly^1^.

High soil strength often limits root propagation and prevents plants to obtain water and other resources available in subsoil^2^. Subsoil strength tends to be naturally high because of the above soil column's weight and internal frictional forces^3^ mostly caused by compacted soil due to agricultural mismanagement^4^.

In recent years, the interest in understanding the mechanisms and prediction of soil tillage performance has increased dramatically because of growing evidence and farmers' concern that the soil structure's quality is adversely affected by agricultural machinery. In assessing the impacts of tillage operations, agricultural engineers seek to predict the effect of soil-tool interaction. Non-inversion of soil has been controlled using tine implements like subsoiler. Subsoiler is an implement that aims at loosening the soil structure and decreasing the bulk density of the subsoil without turning or mixing soil horizons.

In the past two decades, attempts have been made to develop empirical, analytical, and numerical models for soil-tool interactions used in the design of tillage tools to reduce the forces without considering the resulting soil profile^5–7^. Since the machine sizes need to be optimized, the optimum between effort and result needs to be established more intelligently. After tillage, the soil profile is a significant factor; it indicates and shows the outcome of force applied by tillage tools, which provides knowledge about the soil movement and desired disturbance^8^.

Analytical models represent a closed-form mathematical solution to the governing soil mechanics equation subject to the input and output conditions. Numerical models are based on a numerical procedure, such as a discrete element method. Empirical models involve physical experiments and regression equations. Some studies have been conducted on optimizing the tillage process by reducing the tillage forces^9^.

In the analytical approach, equations were developed from a model to predict the effect of tool speed and depth on the total, horizontal, and vertical forces. The model is based on three-dimensional soil wedges and is in the general form of the Reece earth moving Equation. The model was developed by^10^ and an additional term was added^11^ to accommodate the effects of tool speed^6,11–13^. However, most of these researches were based on finding the angle of the failure plane and the effect of the rake angle on soil cutting factors. Likewise, ^14^used an analytical approach to predict soil profile parameters of trough formed by the passage of tines through the soil.

Sun et al.^15^ used the Discrete Element Method to study a bionic subsoiler energy consumption and soil disturbance. Many other researchers used DEM to study subsoiler-soil interactions. With the relative errors of the simulated results, less than 4%, ^16,17^proved that DEM was an effective way of predicting the draft force of subsoilers. ^18^reported that after setting the proper values for the DEM parameters for a soil condition, the profile of the soil failure that depends on the shank's geometry can be satisfactorily simulated under the same soil condition.

This study aims to provide the base for improving deep tillage performance, structure, and working parameters of the non-inversion tool in cohesive soils. This study used an analytical model and a numerical model to determine how much force is needed in tillage to help smart agricultural machinery designers to design a machine according to the needs of the field. Further, the analytical and discrete element models were compared in terms of their advantages and limitations. The suitability of the two models used in the analysis was also determined based on the accuracy of predictions of forces required to cut the soil and the soil furrow profile formed after passage of the subsoiler compared to the experimental data measured using force sensors and soil profilometer respectively.

## Materials and methods

### Experimental materials

The test soil was classified as clay, which is agricultural soil. The average bulk density and cone indexes were 1667.7 kg/m^3^ and 1342 kPa, respectively. Additionally, the moisture contents of the topsoil and subsoil were 10.8% and 11.2%, respectively. The soil physical properties measured at the soil bin are shown in Table 1.

**Table 1:** Soil physical properties measured at the soil bin.

Depth (mm)	Bulk density (kg/m^3^)	Cone index (kPa)
0–50	1463.7	325
50–100	1576.8	401
100–150	1665.4	788
150–200	1718.4	970
200–250	1728.5	1553
250–300	1734.2	2326
300 +	1787.4	3031
Average	1667.7	1342

**Figure 1:**
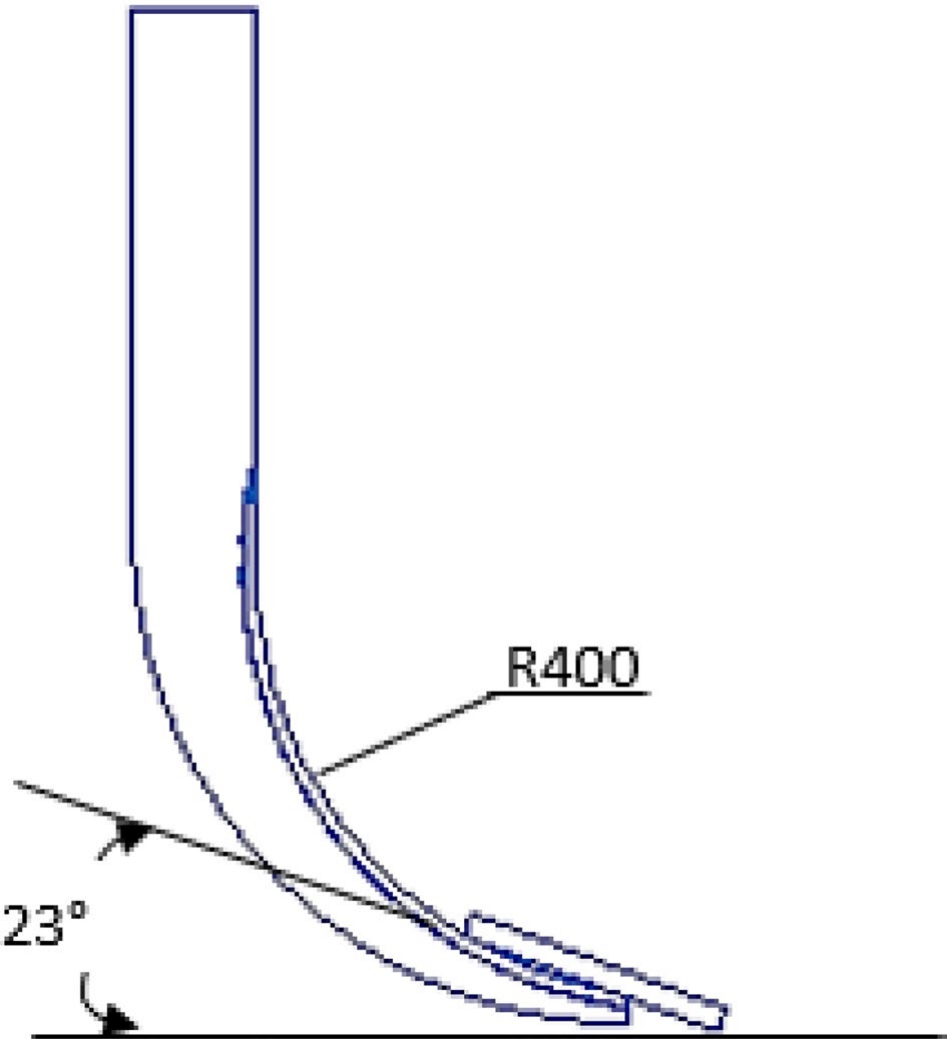
Schematic diagram of the subsoiler shank.

A rectangular blade subsoiler was used in this study. The subsoiler blade formed an angle of 23° with the ground, a circular arc subsoiler shank with a cutting-edge angle of 60° and a thickness of 30 mm. The schematic diagram of the subsoiler shank with a circular arc of 400 mm is shown in Fig. 1.

### Research methods

#### DEM simulation

The discrete element analysis software EDEM 2018 was used in this study to develop a model that considers soil as discrete particles. The same soil properties used by^19^ were used to model the soil-subsoiler interactions. The particles in the model behaved in a linear elastic manner up to a predefined stress, thereafter, the particles experienced plastic deformation. The contact model used was a hysteretic spring contact model integrated with a linear cohesion. The governing equations of the hysteretic spring and linear cohesion contact model are described by^20^.

Firstly, the model's calibration was done by matching the behavior of the angle of repose measured in the laboratory and the one created in the EDEM simulation. In the laboratory, the angle of repose was measured using the funnel filled with soil and raised slowly to form the conical shape of the material heap to minimize the effect of the falling particles. After the heap reached a stable point, the angle of repose was measured by the inverse tangent (arctan) rule at which the average radius of the formed conical shape and the maximum height of the heaped material were measured, and then the angle of repose was determined as the arctan of the maximum height to average radius ratio. Then, the same method of using funnel was simulated in the EDEM. The angle of repose was measured using a protractor tool built-in EDEM Analyst.

The simulation parameters were obtained using an inverse parameterization method. Using the parameters shown in Table 2, the simulation achieved an angle of repose of 36.87° by varying time step, soil-soil coefficient of friction and soil-soil coefficient of rolling friction.

**Table 2:** Basic parameters of the discrete element model.

Parameter	Value
Density of soil particle (kg/m^3^)	2650
Density of steel (kg/m^3^)	7850
Shear modulus of soil (Pa)	5 × 10^7^
Shear modulus of steel (Pa)	7.9 × 10^10^
Poisson's ratio of soil	0.3
Poisson's ratio of steel	0.3
Coefficient of restitution of soil-soil	0.2
Coefficient of static friction of soil-soil	0.45
Coefficient of static friction of soil-steel	0.55
Coefficient of rolling friction of soil-soil	0.13
Coefficient of rolling friction of soil-steel	0.05
Cohesion of the soil (kPa)	10

**Figure 2:**
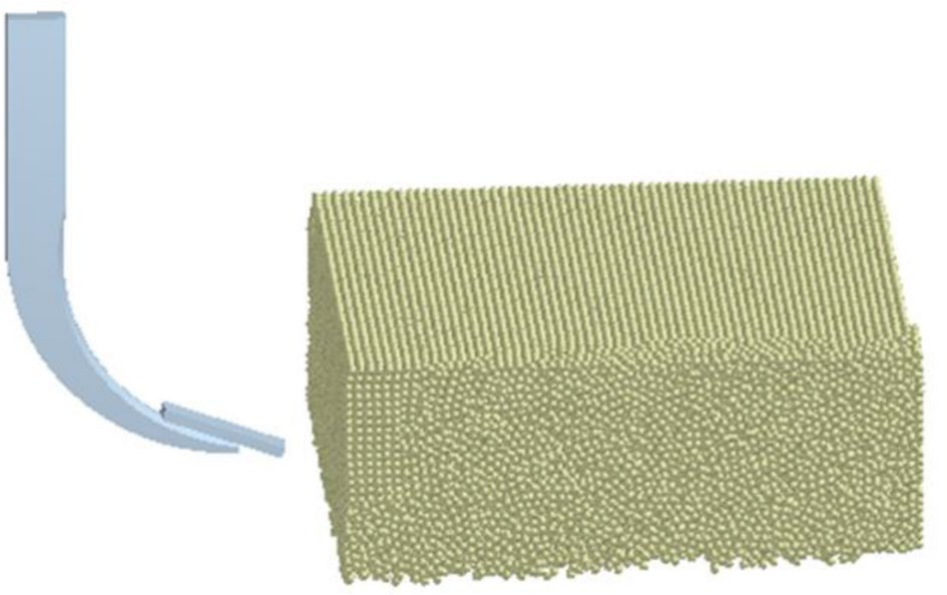
Setup of subsoiler geometry created using Creo Parametric 4.0 software, PTC Inc. (https://www.ptc.com) and the virtual soil bin created using EDEM 2018 software, DEM Solutions Ltd. (https://www.edemsimulation.com/software/).

After calibration of the model using the angle of repose, a soil model was created and compacted to obtain the bulk density, which is the same as that of the soil in the soil bin. A total of 79,824 spherical particles were generated in the simulation to create a virtual soil bin having 1000 mm long × 500 mm wide × 400 mm depth. The subsoiler geometry was made using PTC Creo Parametric 4.0 software^21^ and imported into the EDEM 2018 software to analyze force (Fig. 2). Afterward, the calibration parameters obtained were used to conduct the simulation experiments to predict the horizontal, vertical cutting forces and the soil profile. The accuracy and efficiency of the model were tested.

#### Analytical equation

The analytical equation (Eq. 1) developed by^11^ was used to obtain horizontal and vertical forces. The parameters used in the equation were obtained from the soil bin experiments and the laboratory tests.

1 $$P = \left( {p{\text{g}}d^{2} N_{\gamma } + CdN_{c} + C_{a} dN_{ca} + qdN_{q} + \rho S^{2} dN_{a} } \right)W$$

where $p$, is the initial soil density (kg/m^3^), ${\text{g}}$ is the gravity (m/s^2^), $d$ is the operating depth (m), $C$ is the soil cohesion (kPa), $C_{a}$ is the soil adhesion (kPa), $q$ is the surcharge pressure (kPa), $W$ is the tool width (m), $N$ is the dimensionless factors relating to $\Phi$ (friction angle), $\delta$ (soil-metal friction angle) and $\alpha$ (the rake angle) and $S$ is the tool speed (m/s).

Using the MATLAB R2020a (The MathWorks Inc., Natick, MA), Eqs. (1–9) were solved for all terms to obtain total force ($P$). The soil parameters were drawn from Table 1. The tool width for Eq. (1) was taken as the effective width of the blade, 260 mm, and the rake angle was 16°. Similarly, the horizontal and vertical components of the total force were calculated using Eqs. (12) and (13) ^11^. All dimensionless cutting factors were obtained from the work presented by^6^.

2 $$F_{x} = P\sin \left( {\alpha + \delta } \right) + C_{a} + C_{a} dW\cot \alpha$$

3 $$F_{y} = P\cos \left( {\alpha + \delta } \right) - C_{a} dW$$

4 $$N_{{\upgamma }} = \frac{{\frac{r}{2d}\left\{ {1 + \left. {\frac{2s}{{3w}}} \right\}} \right.\sin \left( {\beta + \varphi )} \right.}}{{\sin \left( {\alpha + \beta + \delta + \varphi )} \right.}}$$

5 $$N_{c} = \frac{{\frac{\cos \varphi }{{\sin \beta }}\left\{ {1 + \left. \frac{s}{w} \right\}} \right.}}{{\sin \left( {\alpha + \beta + \delta + \varphi )} \right.}}$$

6 $$N_{ca} = \frac{{ - \cos \left( {\alpha + \beta + \varphi )} \right.}}{{\sin \alpha \sin \left( {\alpha + \beta + \delta + \varphi )} \right.}}$$

7 $$N_{q} = \frac{{\frac{r}{d}\left\{ {1 + \left. \frac{s}{w} \right\}} \right.\sin \left( {\beta + \varphi )} \right.}}{{\sin \left( {\alpha + \beta + \delta + \varphi )} \right.}}$$

8 $$S = d\sqrt {(\cot \beta )^{2} + 2\cot \alpha \cot \beta }$$

9 $$N_{a} = \frac{{\tan \beta + \cot \left( {\beta + \varphi )} \right.}}{{(\cos \left( {\alpha + \delta } \right) + \sin \left( {\alpha + \delta )\cot \left( {\beta + \varphi } \right))(1 + \tan \beta \cot \alpha )} \right.}}$$

where $N_{a}$ is a dimensionless factor for soil inertia effect, is a function of $\alpha$, $\delta$, $\varphi$ and $\beta$, and is given by Eq. (9). Other notations, as mentioned previously.

Willatt and Willis^14^ developed an equation to predict the width of furrow for curved and plain tines, as shown by Eq. (10). The furrow disturbed by both tines were roughly trapezoidal.

10 $$Wf = \left( {2.42d + W} \right) \times k$$

where $Wf$ is the width of the furrow (m), d is the operating depth (m), $W$ is the tine width (m), and $k$ is a constant and which is equal to 0.0254.

#### Experimentation for verification of the model

**Figure 3:**
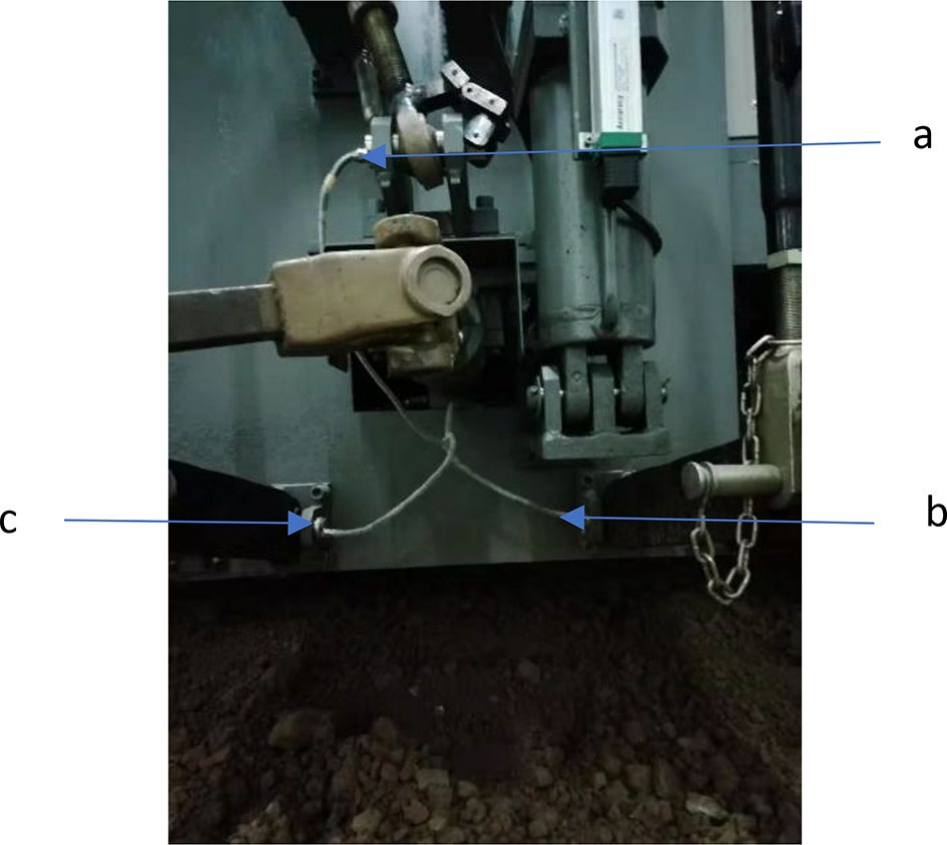
Sensors for force measurement at the three-point linkages behind the soil bin trolley (**a**) Top link (**b**) Right lower link (**c**) Left lower link.

Verification tests were conducted under controlled conditions using an indoor soil bin trolley set up with a PLC controller. All the tests were conducted between September 2019 and February 2020 at Nanjing Agricultural University, Agricultural Machinery Testing Center, Nanjing, Jiangsu Province, China. A trolley developed by the Nanjing Agricultural University was used to pull the implement in the soil bin. Forces were measured by sensors placed at three points linkages, as shown in Fig. 3. After that, the measured forces were resolved to obtain the force's vertical and horizontal (draught) components. Equations (11) to (20) show how forces were distributed in the trolley rear lifting links. The resolving of force followed the method of force calculation provided by^22^.

The diagrams for the links of the soil bin trolley are presented in Fig. 3. The technical specification for the KMB 40 force sensor used to measure cutting forces in the soil bin is shown in Table 3. The angles were varying according to the depth of the implement.

**Table 3:** Technical specification of KMB 40 force sensor.

Items	Technical parameters
Measurement range (MPa)	− 0.1–70
Supply voltage (V)	DC 10–24
Full-scale output (mV)	DC 70–110
Corresponding time (10–90%) (ms)	≤ 1
Operating temperature range	− 35 °C to + 85 °C
Plug connection	3-pin connector with single-wire seal
Load resistance	> 10 kΩ
Standard overload range	± 220 kN
Supply current Isup	< 50 mA

**Figure 4:**
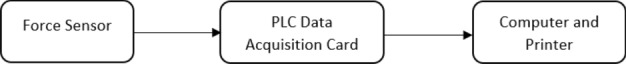
Flow chart of the data acquisition process.

**Figure 5:**
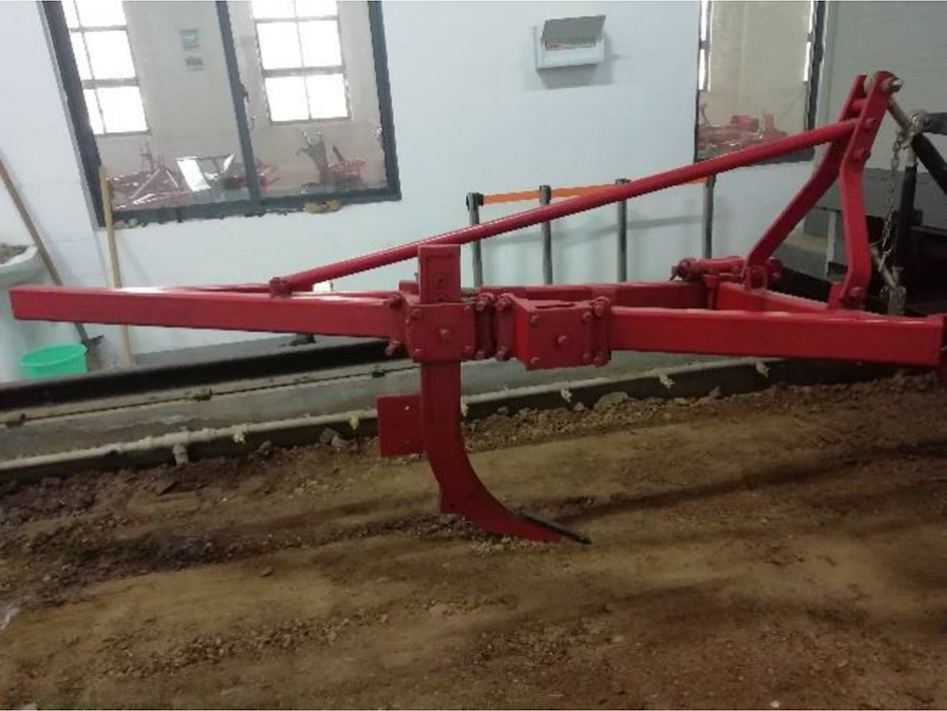
Subsoiler attached to the soil bin trolley.

Data acquisition for cutting forces at three-point linkages done by a PLC controller on the soil bin trolley is shown in the flow chart (Fig. 4). The force sensor comprises a bearing bolt that encounters shear stress and records as elongation and evaluated. The measurement principle is based on the measurement of mechanical elongation using strain gauges, which are wired to form a bridge circuit within the force sensor. When not under load, the bridge is in equilibrium while when a force is applied, the output signal of the DMS (database management system) bridge changes, either positive or negative, according to the direction of the force applied. This magnitude of the bridge voltage is proportional to the force used. The subsoiler attached to the soil bin trolley is shown in Fig. 5.

**Figure 6:**
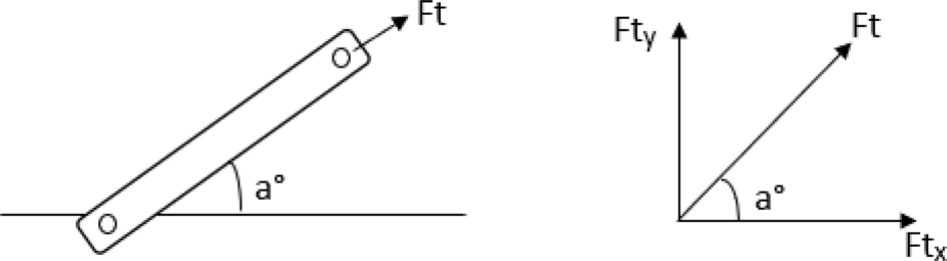
Diagrams showing the direction of the force in the top link.

**Figure 7:**
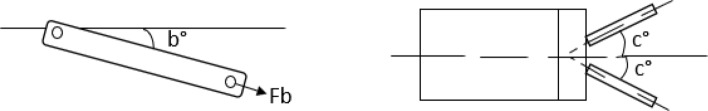
Diagrams showing the direction of the force in the lower/bottom links.

The diagrams showing the direction of the force in the top and lower/bottom links are shown in Figs. 6 and 7 respectively. The same procedure of resolving forces in the top and lower/bottom links of tractor was used by^23^.

11 $$Ft_{x} = Ft.Cosa$$

12 $$Ft_{y} = Ft.Sina$$

where $Ft$ is the force exerted only in the direction of the axis of the upper arm,$Ft_{x}$, $Ft_{y}$ are the horizontal and vertical components of $Ft$, $a$ is the angle between the upper link and the horizontal line.

13 $$Fbl_{x} = Fblh.Cosb.CosC - Fblv.Sinb.CosC$$

14 $$Fbr_{x} = Fbrh.Cosb.CosC - Fbrv.Sinb.CosC$$

15 $$Fbl_{y} = Fblh.Sinb + Fblv.Cosb$$

16 $$Fbr_{y} = Fbrh.Sinb + Fbrv.Cosb$$

Computation of the horizontal and vertical forces

17 $$F_{x} = Ft_{x} + Fbl_{x} + Fbr_{x}$$

18 $$F_{y} = Ft_{y} + Fbl_{y} + Fbr_{y}$$

By substituting Eqs. (11), (13) and (14) into (17).

19 $$F_{x} = Ft.Cosa + Fblh.Cosb.Cosc + Fbrh.Cosb.Cosc$$

Next, Eqs. (12), (15), and (16) into (18).

20 $$F_{y} = Ft.Sina + Fblh.Sinb + Fbrh.Sinb$$

where $Fblh$, $Fbrh$ are the forces in the left and right lower hitch points axis respectively, $F_{x}$ is the horizontal (draught) force, $F_{y}$ is the vertical force, $Fbl_{x}$, $Fbl_{y}$ are the horizontal and vertical forces on the left lower link, $Fbr_{x} , Fbr_{y}$ are the horizontal and vertical forces on the right lower link, $b$ and $c$ are angles of the lower arms. Subsequently, the MATLAB programming language was used to solve all terms to obtain forces.

Further, the soil furrow profile formed after the subsoiler passage was measured using the soil profilometer. The penetrometer was pushed into the soil by hand at a speed of approximately 0–2 m/s as per ASABE standards^24^.

#### Statistical analysis

The Taguchi method developed by^25^, which uses an orthogonal array to optimize the entire parameter space with fewer experiments, was used to design the experiments. Orthogonal arrays are a special standard experimental design that requires only a small number of experimental trials to find the main factors affecting output. Before selecting an orthogonal array, the minimum number of experiments conducted was fixed based on the formula shown in Eq. (21).

21 $$N Taguchi = 1 + NV\left( {L - 1} \right)$$

where $N Taguchi$ = number of experiments to be conducted, $NV$ = number of parameters, $L$ = number of levels. In this case, 27 experiments were supposed to be conducted but using the L9 orthogonal array, only 9 experiments were sufficient to optimize the parameters.

The relative error method was used to calculate the percentage error and compare the simulated and analytical results with the soil bin's measured values, as shown in Eq. (22).

22 $$Relative Error = \frac{Measured value - Predicted value}{{Measured value}} \times 100\%$$

Additionally, ANOVA test was performed to determine the differences between the soil bin experiment, analytical method, and the DEM simulation data.

## Results

### Cutting forces

#### Validation of the developed models on cutting forces

The statistical performance of the forces measured during the tillage experiment in the soil bin, and one estimated by the analytical, and the numerical models are shown in Table 4. The results showed a close relationship between the numerical and experimental values, with the relative errors of the predicted results being 4.44 and 4.01% for horizontal and vertical forces.

**Table 4:** The forces measured during the tillage experiments in the soil bin, analytical approach, and the numerical model.

Speed (km/h)	Depth (m)	Analytical approach (N)		DEM model (N)		Experiment/		Relative error (%)			
						Soil bin (N)		Analytical		DEM	
		H	V	H	V	H	V	H	V	H	V
1	0.15	1201.87	478.28	1283.54	480.43	1235.24	490.22	2.78	2.50	3.76	2.04
1	0.2	1809.24	719.98	1951.00	720.32	1905.15	740.51	5.30	2.85	2.35	2.80
1	0.3	3334.11	1326.80	3588.41	1328.61	3381.81	1390.34	1.43	4.79	5.76	4.65
2	0.15	1203.49	478.92	1295.28	485.12	1296.55	500.23	7.73	4.45	0.10	3.11
2	0.2	1811.39	720.84	1985.00	721.90	1934.67	750.91	6.81	4.17	2.54	4.02
2	0.3	3337.34	1328.09	3791.89	1332.33	3389.01	1401.90	1.55	5.56	10.62	5.22
2.5	0.15	1204.69	479.41	1296.58	491.41	1312.11	510.50	8.92	6.49	1.20	3.88
2.5	0.2	1813.01	721.48	2021.00	723.01	1941.32	760.45	7.08	5.40	3.94	5.18
2.5	0.3	3339.77	1329.06	3894.50	1340.34	3417.52	1410.11	2.33	6.10	12.25	5.21
Average relative error (%)								4.88	4.70	4.44	4.01

H, V- Indicates horizontal and vertical forces respectively.

Furthermore, the ANOVA-test proved no significant difference between the analytical model, DEM simulation and soil bin experimental results at a 0.05% level, as shown in Table 5. The *p*-values were 0.9396 and 0.9696 for the horizontal and vertical forces, respectively, which were non-significant, i.e. $p > 0.05$.

**Table 5:** Summary of ANOVA for assessing the statistical significance between Analytical model, DEM simulation, and soil bin experiment results.

	Sum of squares	df	Mean square	F-Stat	*P*-value
**For horizontal force**					
Between groups	117,351.37	2	58,675.68	0.063	0.9396^ns^
Within groups	22,529,201.59	24	938,716.73		
Total	22,646,552.96	26			
**For vertical force**					
Between groups	9269.97	2	4634.99	0.031	0.9696^ns^
Within groups	3,594,751.79	24	149,781.32		
Total	3,604,021.76	26			

**, ^ns^- Indicates significant at 0.05% level and non-significant, respectively.

**Figure 8:**
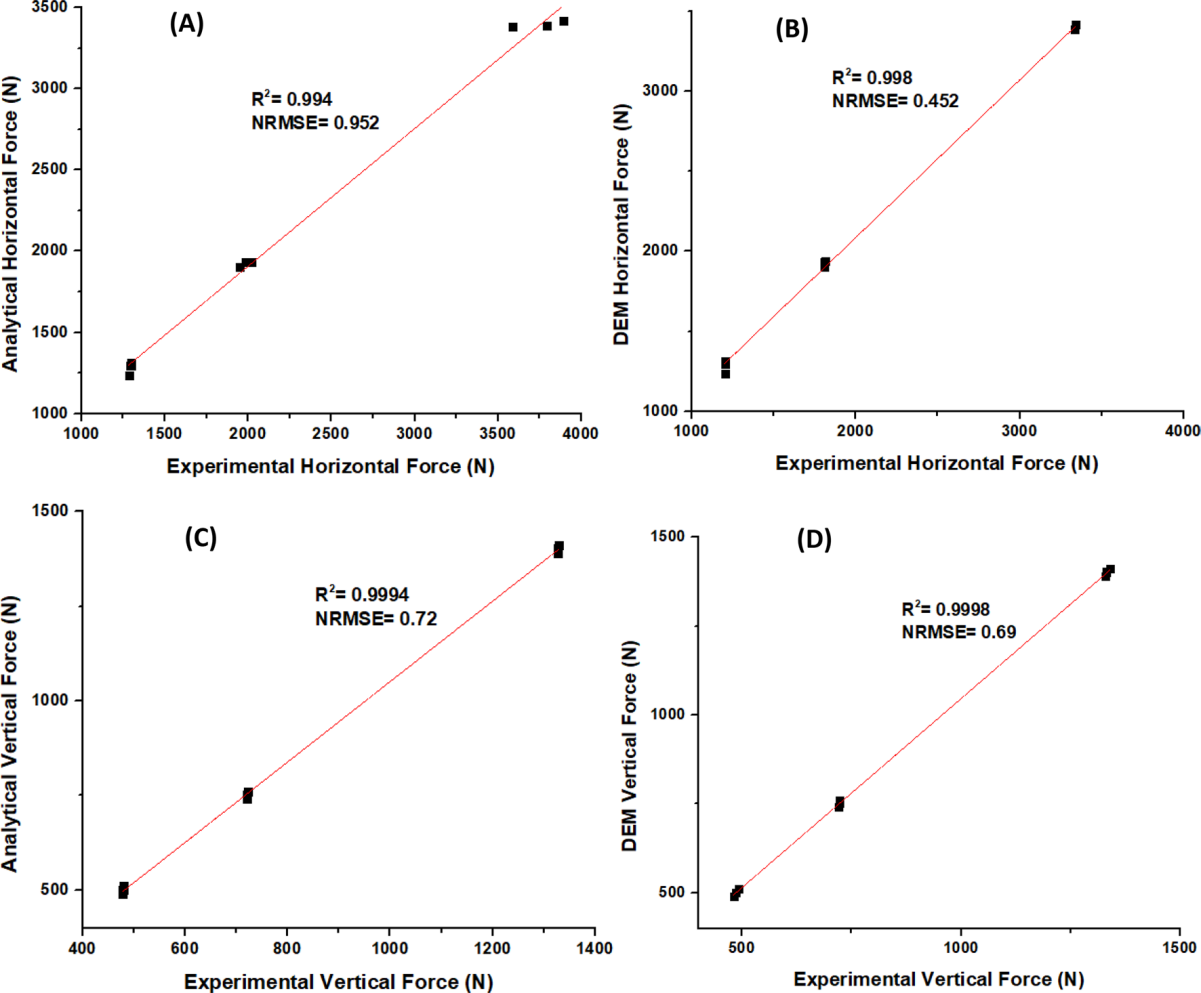
The linear regression plots for the predicted against measured horizontal forces (**A**) and (**B**) and vertical forces (**C**) and (**D**).

The results also indicate that DEM predicted results with the best regression of 0.9998 $R^{2}$ at a normalized $RMSE$ of 0.69 for vertical force followed by the DEM model with 0.998 $R^{2}$ and a normalized $RMSE$ of 0.452 for horizontal force, while the analytical model had the lowest $R^{2}$ of 0.994, at a normalized $RMSE$ of 0.952 (Fig. 8). However, all three regression results gave a p-value < 0.0001.

#### Prediction of cutting forces

**Figure 9:**
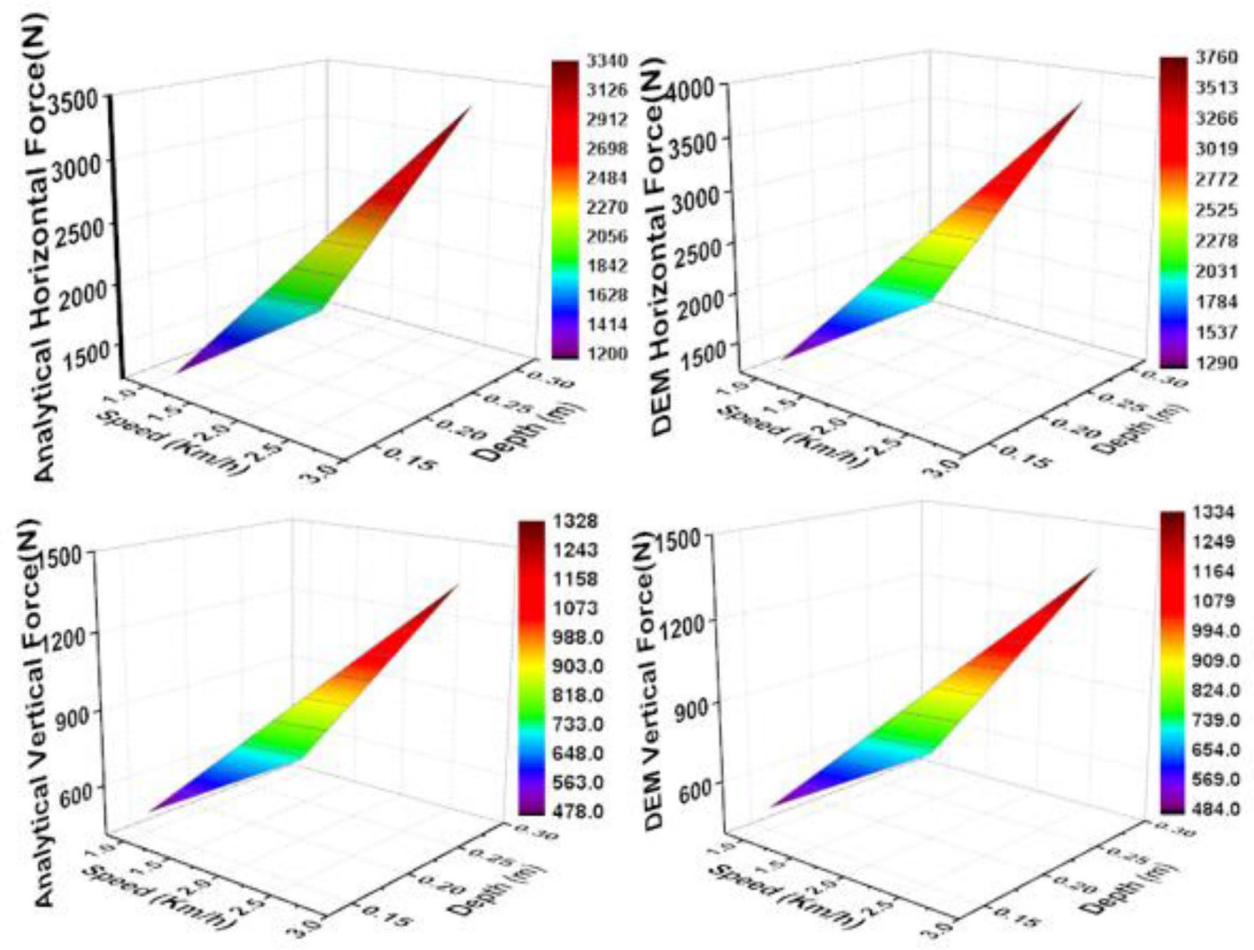
The prediction of cutting forces of the subsoiler during tillage operations (**A**) Analytical horizontal force (**B**) DEM horizontal force (**C**) Analytical vertical force (**D**) DEM vertical force.

It was found that both horizontal and vertical cutting forces increased as tillage depth was increased from 0.15 to 0.30 m (Fig. 9). Any increment of tillage depth leads to the increase of soil volume cut, dispersed, and moved. Therefore, a higher cutting force is required to break higher soil volume. ^26^ obtained the same trend of results in their study of kinematic parameters of chisel ploughs.

The results also show that an increment of operating speed from 1 to 2.5 km/h resulted in cutting force growth. This is because soil particles intend to gain higher acceleration as operating speed increases. The higher acceleration of particles increased normal loads acting on the tillage tool. When the normal load increases, the frictional force and thereby, the cutting force increases^27^.

Similar results were obtained by^28^ after using the ASABE standard Equation ^24^ to measure the draft forces of a subsoiler.

### Soil furrow profile

#### Validation of the developed models on soil profile formation

The furrow widths' statistical performance measured after the subsoiler passage in the soil bin, calculated by analytical approach and the numerical model estimate are shown in Table 6. The results showed a close relationship between the numerically predicted values and the experimental ones, with the relative errors of the prediction of 10.83 and 64.38% for numerical and analytical respectively.

**Table 6:** Dimensions of the furrow width (Wf) formed after the passage of the subsoiler at a speed of 2.5 km/h.

Depth (m)	Experiment	Analytical model	DEM model	Relative error (%)	
				Analytical	DEM
0.10	0.30	0.07	0.25	76.13	16.67
0.15	0.32	0.10	0.29	68.00	9.38
0.20	0.35	0.13	0.31	61.97	11.43
0.25	0.40	0.16	0.36	59.05	10.00
0.30	0.45	0.19	0.42	56.76	6.67
Average relative error (%)				64.38	10.83

**Figure 10:**
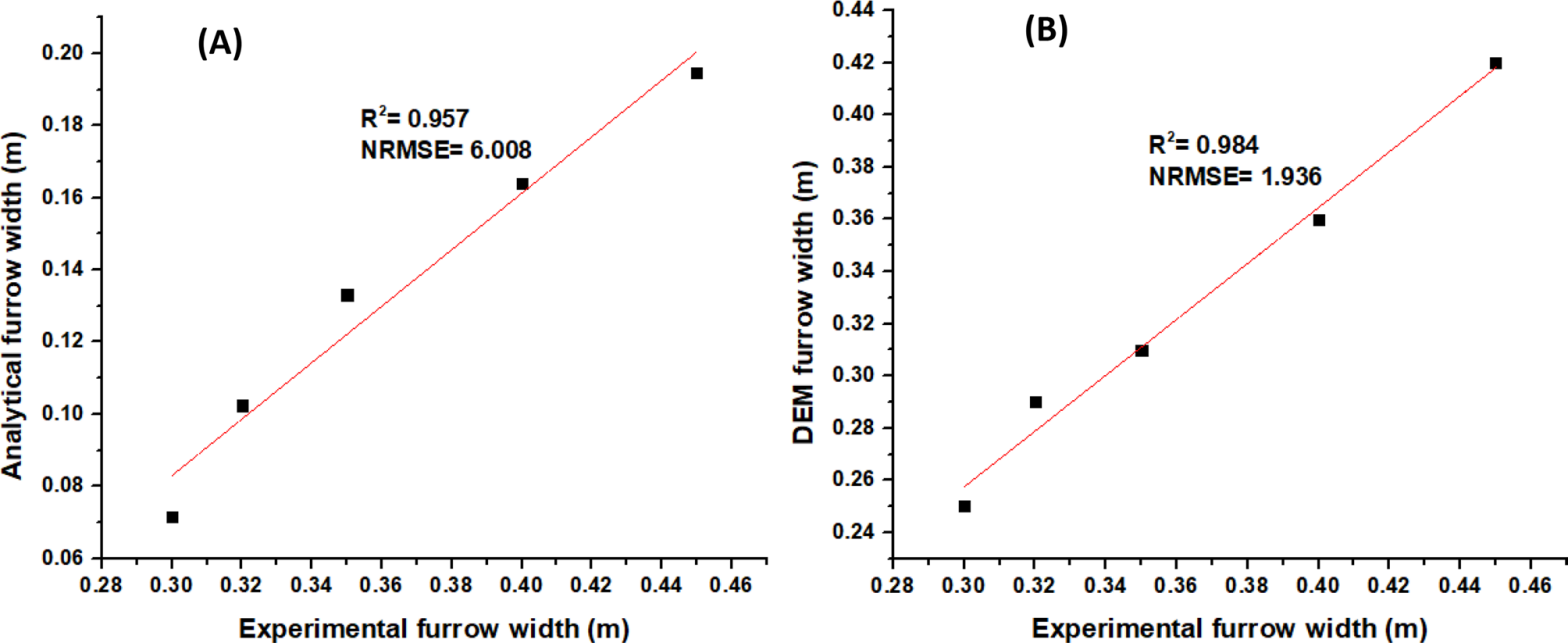
Comparison between predicted (**A**) Analytical model (**B**) DEM model and measured width of furrow for a subsoiler.

The results also indicate that DEM predicted the results with the best regression of 0.984 $R^{2}$ at a normalized $RMSE$ of 1.936 while the analytical model had the lowest $R^{2}$ of 0.957, at a normalized $RMSE$ of 6.008 (Fig. 10). However, all three regression results gave a *p*-value < 0.0001.

#### Prediction of the soil profile

**Figure 11:**
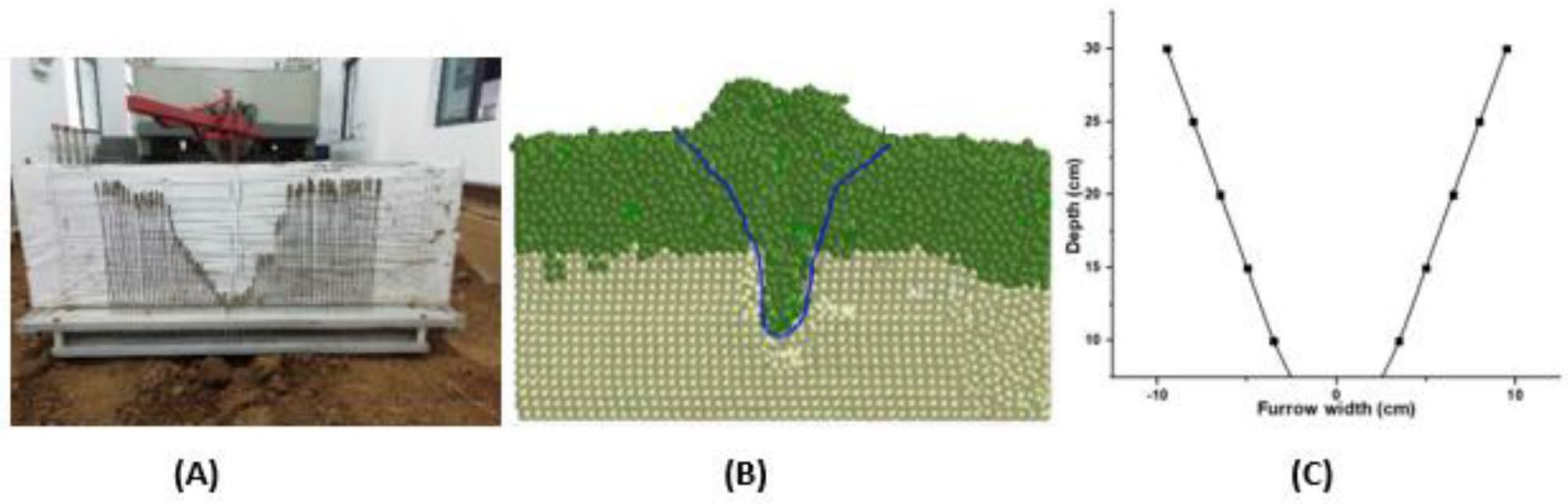
The furrow profile formed after passage of subsoiler during tillage operations (**A**) Soil bin experiment (**B**) DEM model created using EDEM 2018 software, DEM Solutions Ltd. (https://www.edemsimulation.com/software/) (**C**) Analytical model.

The DEM and the analytical models' results were compared with experimental results to study the soil profile shape formed after passage of subsoiler. As depicted in Fig. 11, the shape obtained in the DEM simulation gave results that are more similar to the experimental ones as compared to the analytical approach. However, it was challenging to show profile appearance dynamics in the analytical approach.

After the passage of the subsoiler, the disturbed particles fall back to the furrow. Therefore, the formed furrow profile obtained in the DEM was measured at the edge of the disturbed particles separated by different colors. The analytical profile was not able to show the dynamics of the formed furrow profile shape.

However, DEM showed that the subsoiler did not turn the soil and hence maintaining the soil structure.

## Discussion and conclusions

The results from this study indicated that there was no turning of soil caused by the passage of the subsoiler at the depth below the top layer of soil. This was a result of the shape of the subsoiler. To calculate the two models' accuracies, the cutting forces and the soil furrow profiles formed after the subsoiler passage were measured and compared with the experimental data obtained in the soil bin data in terms of relative error and R squared.

Depending on the analysis, the vertical force's prediction accuracy was higher compared with that of the predicted draught force. Generally, the values of calculated RE obtained were less than 5%, i.e. 95% accuracy was achieved in verifying the tested models. Likewise, the ANOVA test showed no statistically significant differences between the analytical model, DEM simulation, and soil bin experiment results at 0.05% level, as shown in Table 5.

Both the horizontal and vertical forces increased with the increase in depth of cut and the operating speed. The same results of tillage forces were obtained by^29^. However, the predicted values of force obtained in the DEM simulation were higher than the analytical ones, which is closer to the experimental results. This can be attributed to the closeness of the characteristics between the experimental and the simulation parameters used.

The Wf increased linearly with the operating depth. Identical results of AM have been found by^8,30^ in their study of cutting forces and soil disturbance.

This result reveals that tine implements like subsoiler do not turn cohesive soil, hence maintaining the soil layers of the soil profile. As was observed, the simulation model can be utilized as a tool to examine the induced changes in the soil profile and prediction of the furrow formed after the passage of the subsoiler. The formed profiles' shapes were trapezoidal as observed by^17,31,32^.

This means that to maintain soil nutrients in deep tillage, especially for the fields with less depth of organic soil, it is advisable to use tine implements (subsoiling) that do not turn the soil's bottom layer, which does not contain good organic needed for crop growth. It was also observed that the subsoiling operation conserved soil due to the backfill of the topsoil (organic soil) to the subsoil, which provides nutrients for the deep roots (Fig. 11B).

In conclusion, the findings of this study provide the base for improving deep tillage performance, structure, and working parameters of the non-inversion tool in cohesive soils. The improvement of deep tillage by utilizing the developed model can also lead to the evolution of the tillage operations which is another way for the development of the agricultural machinery manufacturing sector. To avoid the expensive field testing and obtain a prediction of the cutting forces and soil profiles of different tillage implements, it is thus important to adopt the model suggested in this study. Future work will need to develop an improved contact model to improve the results and develop an improved calibration procedure that considers the effects of all the micro-properties parameters of soil mechanical properties and use closer to actual particle sizes.

## Nomenclature

$Ft$: Force exerted only in the direction of the axis of the upper arm (N)
$Ft_{x}$: Horizontal components of $Ft$ (N)
$Ft_{y}$: Vertical components of $Ft$ (N)
$Fblh$: Forces in the left lower hitch points axis (N)
$Fbrh$: Forces in the right lower hitch points axis (N)
$Fbl_{x}$: Horizontal forces on the left lower link (N)
$Fbl_{y}$: Vertical forces on the left lower link (N)
$Fbr_{x}$: Horizontal forces on the right lower link (N)
$Fbr_{y}$: Vertical forces on the right lower link (N)
$F_{x}$: Horizontal (draught) force (N)
$F_{y}$: The vertical force (N)
$a$: The angle between the upper link and the horizontal line (°)
$b$: The angle of the lower arms with the vertical axis (°)
$c$: The angle of the lower arms with the horizontal axis (°)
$$P$$: Total force (N)
$p$: Initial soil density (kg/m^3^)
$${\text{G}}$$: Gravity (m/s^2^)
$$d$$: Operating depth (m)
$C$: Soil cohesion (kPa)
$C_{a}$: Soil adhesion (kPa)
$q$: Surcharge pressure (kPa)
$$W$$: Tool width (m)
$$\Phi$$: Friction angle (°)
$\delta$: Soil-metal friction angle (°)
$$\alpha$$: Rake angle (°)
$$S$$: Tool speed (m/s)
$N$: Dimensionless factors relating to $\Phi , \delta {\text{and}} \alpha$
$$r$$: Rupture distance (m)
$Wf$: Width of the furrow (m)
$k$: Constant which is equal to 0.0254
